# An alternative polysaccharide uptake mechanism of marine bacteria

**DOI:** 10.1038/ismej.2017.26

**Published:** 2017-03-21

**Authors:** Greta Reintjes, Carol Arnosti, Bernhard M Fuchs, Rudolf Amann

**Affiliations:** 1 Department of Molecular Ecology, Max Planck Institute for Marine Microbiology, Bremen, Germany; 2 Department of Marine Sciences, University of North Carolina-Chapel Hill, Chapel Hill, NC, USA

## Abstract

Heterotrophic microbial communities process much of the carbon fixed by phytoplankton in the ocean, thus having a critical role in the global carbon cycle. A major fraction of the phytoplankton-derived substrates are high-molecular-weight (HMW) polysaccharides. For bacterial uptake, these substrates must initially be hydrolysed to smaller sizes by extracellular enzymes. We investigated polysaccharide hydrolysis by microbial communities during a transect of the Atlantic Ocean, and serendipitously discovered—using super-resolution structured illumination microscopy—that up to 26% of total cells showed uptake of fluorescently labelled polysaccharides (FLA-PS). Fluorescence *in situ* hybridisation identified these organisms as members of the bacterial phyla *Bacteroidetes* and *Planctomycetes* and the gammaproteobacterial genus *Catenovulum*. Simultaneous membrane staining with nile red indicated that the FLA-PS labelling occurred in the cell but not in the cytoplasm. The dynamics of FLA-PS staining was further investigated in pure culture experiments using *Gramella forsetii*, a marine member of *Bacteroidetes*. The staining patterns observed in environmental samples and pure culture tests are consistent with a ‘selfish’ uptake mechanisms of larger oligosaccharides (>600 Da), as demonstrated for gut *Bacteroidetes*. Ecologically, this alternative polysaccharide uptake mechanism secures substantial quantities of substrate in the periplasmic space, where further processing can occur without diffusive loss. Such a mechanism challenges the paradigm that hydrolysis of HMW substrates inevitably yields low-molecular-weight fragments that are available to the surrounding community and demonstrates the importance of an alternative mechanism of polysaccharide uptake in marine bacteria.

## Introduction

Marine microbial communities are responsible for processing an estimated half of the organic carbon annually produced in the ocean (Azam and Malfatti, 2007). It is generally assumed that the high-molecular-weight (HMW) fraction of this organic matter is hydrolysed initially by extracellular enzymes to sizes <600 Da (Weiss *et al.*, 1991) for transport into the cell (Arnosti, 2011). The conditions under which the production of an extracellular enzyme might be energetically beneficial, the extent to which diffusive loss of hydrolysed products limits the utility of extracellular enzyme production and scenarios under which non-enzyme-producing organisms may benefit from the activities of enzyme producers have been considered in recent models (for example, Vetter *et al.*, 1998; Allison, 2005; Traving *et al.*, 2015). These models assume that hydrolysis occurs in the extracellular environment and that hydrolysis products therefore are available to the wider microbial community. Models consequently typically express substrate availability in terms of monomer production and transport. Field measurements have likewise measured carbohydrate metabolism by microbial communities in the ocean via production and uptake of monosaccharides (for example, Rich *et al.*, 1996). With few exceptions, moreover, investigations of carbohydrate dynamics have focussed primarily on enzymatic hydrolysis of glucose-containing substrate proxies (MUF-α- and β-glucose; Zaccone *et al.*, 2012; Kellogg and Deming, 2014); glucose dynamics have also been used as a representation of polysaccharide metabolism in general (Christian and Karl, 1995; Piontek *et al.*, 2014).

Different marine heterotrophs, however, specialise in uptake of low-molecular-weight and HMW substrates (for example, Cottrell and Kirchman, 2000; Elifantz *et al.*, 2005, 2007). Natural microbial communities in surface ocean waters also exhibit substrate preferences. Differences in the spectrum of polysaccharides hydrolysed (Arnosti *et al.*, 2005, 2012) and a latitudinal gradient in enzyme activities (Arnosti *et al.*, 2011) that parallels large-scale patterns in microbial biogeography have been demonstrated (for example, Baldwin *et al.*, 2005; Fuhrman *et al.*, 2008; Wietz *et al.*, 2010). The importance in marine systems of an alternative substrate uptake mechanism, known for gut bacteria (*Bacteroidetes*), has been suggested by metagenomic data from coastal ocean waters (Teeling *et al.*, 2012, 2016) and oceanic provinces in the North Atlantic (Gómez-Pereira *et al.*, 2012). With this mechanism, polysaccharides are bound to the outer membrane, partially hydrolysed, and transported as larger oligosaccharides (>600 Da) into the periplasm using TonB-dependent outer membrane receptors/transporters (Cho and Salyers, 2001), a mechanism homologous to the starch utilisation system (sus-like) (D’Elia and Salyers, 1996). Direct evidence of the manner in which individual bacteria in the ocean take up HMW polysaccharides, however, is still lacking.

To examine the links between activities and communities across broad spatial scales, we incubated natural microbial communities of five distinct oceanic provinces—Northern Temperate, Northern Gyre, Equatorial, Southern Gyre and Southern Temperate (Longhurst *et al.*, 1995)—of the Atlantic Ocean (Supplementary Figure S1) with specific fluorescently labelled polysaccharides (FLA-PS). These polysaccharides, laminarin, xylan and chondroitin sulphate, were selected because they are present in large quantities in the ocean and/or enzymes that hydrolyse these polysaccharides are widely distributed among marine bacteria. For example, the production of laminarin, an energy storage product of diatoms, has been estimated at 5–15 billion metric tons annually (Alderkamp *et al.*, 2007). Xylan is a major component of red and green algae (Lahaye *et al.*, 1993; Usov, 2011) and thus widely present in the ocean, and chondroitin sulphate, which is commercially derived from shark cartilage, is rapidly and readily hydrolysed across broad ranges of ocean waters (Arnosti, 2011) and by a diverse array of marine bacterial isolates (Wegner *et al.*, 2013; Xing *et al.*, 2015). These polysaccharides also differ in chemical composition: laminarin is a glucose polysaccharide, xylan a polymer of xylose (a pentose rather than hexose sugar), and chondroitin sulphate is a sulphated polymer of *N*-acetylgalactosamine and glucuronic acid. These substrates thus provide the opportunity to probe the activities of a wider range of enzymes (Arnosti, 2003).

We subsampled the incubations at sea and serendipitously observed, using epifluorescence microscopy, that up to 26% of the individual bacterial cells bound the FLA-PS. Pursuing these observations, we combined FLA-PS staining with single-cell identification by fluorescence *in situ* hybridisation (FISH) (Amann *et al.*, 1995) and super-resolution light microscopy to visualise the uptake of FLA-PS by individual bacterial cells in natural communities in surface ocean waters.

## Materials and methods

### Sampling and substrate incubations

Seawater samples were collected aboard the RRV *James Cook* during the Atlantic Meridional Transect 22 cruise from Southampton, UK, to Punta Arenas, Chile, from 10 October to 24 November 2012. In five different oceanic provinces (Supplementary Figure S1) at solar noon, triplicate 20 litre seawater samples were collected from 20 m depth using a Niskin rosette with an attached Sea Bird CTD (Sea Bird Electronics Inc., Bellevue, WA, USA). From each triplicate, subsamples of 500 ml were added to sterile glass bottles and incubated with one of the three FLA-PS, laminarin, chondroitin sulphate and xylan (nine bottles in total), for a total of 12–18 days. In addition, a treatment control, consisting of 500 ml seawater in a sterile glass bottle without a FLA-PS, as well as killed controls, consisting of 50 ml autoclaved seawater with one of the three FLA-PS, were incubated under the same conditions. All bottles were incubated at room temperature (RT) in the dark and sampled at regular time points (typically at 30 min, 1, 3, 6, 12 and 18 days). At each time point, samples for FISH analysis, measurement of extracellular enzyme activities and DNA analysis were collected. For FISH, 20 ml of water was filtered through a 47 mm (0.2 μm pore size) polycarbonate filter, applying a gentle vacuum of <200 mbar. After drying, the filters were stored at −20 °C until further analysis. For DNA analysis, 10 ml was filtered through a 0.2 μm pore size polycarbonate filter using a Whatman 420200 Swin-Lok reusable filter holder (Sigma-Aldrich Chemie GmbH, Munich, Germany).

### FLA-PS synthesis and measurement of extracellular enzymatic activity

Three polysaccharides (laminarin, xylan, chondroitin sulphate) obtained from Sigma-Aldrich (Munich, Germany) were fluorescently labelled with fluorescein amine (Sigma-Aldrich; isomer II) as described in Arnosti (2003). The FLA-PS solutions are free of monosaccharides or oligosaccharides, due to the fact that they are repeatedly injected onto standardised gel permeation chromatography systems as part of the labelling procedure; any low-molecular-weight carbohydrates are thereby removed during purification. Average-molecular weights of fluorescently labelled laminarin, xylan and chondroitin sulphate are 6000, 9000 and >50 000 daltons, respectively. A single polysaccharide was added at a concentration of 1.75 μmol monomer-equivalent to each 500 ml water sample; each polysaccharide was incubated in triplicate, plus one killed control, as described above.

### Substrate staining, FISH and epifluorescence microscopy

For all the time points, the cells were filtered as described and counter-stained with 4',6-diamidino-2-phenylindole (DAPI) and nile red and subsequently mounted using a Citifluor/VectaShield (4:1) mounting solution. Substrate-stained cells were visualised and enumerated using a fully automated microscope imaging system, described in detail by Bennke *et al.* (2016), on a Zeiss AxioImager.Z2 microscope stand (Carl Zeiss MicroImaging GmbH, Göttingen, Germany) with a cooled charged-coupled-device (CCD) camera (AxioCam MRm; Carl Zeiss) and a Colibri LED light source (Carl Zeiss) with three light-emitting diodes (UV-emitting LED, 365±4.5 nm for DAPI; blue emitting LED, 470±14 nm for FLA-PS 488; red-emitting LED, 590±17.5 nm for the tyramide Alexa 594, FISH), combined with the HE-62 multifilter module (Carl Zeiss). This module consists of a triple emission filter TBP 425 (±25), 527 (±27), LP 615, including a triple beam splitter of TFT 395/495/610. All automatic cell counts were validated using manual cell counting. Briefly automated cell counting was carried out by initially acquiring images (using a 63 × magnification and 1.4 numerical aperture oil emersion plan apochromatic objective (Carl Zeiss)), at selected wavelengths (DAPI, FLA-PS, FISH), of a previously defined set of coordinates consisting of a minimum of 46 fields of view on each sample filter (Bennke *et al.*, 2016). Subsequently, the images were imported into the ACMETOOL2.0 (http://www.technobiology.ch/index.php?id=acmetool) image analysis software. From the images, cells were deemed ‘substrate stained’ if they showed a positive signal in both the DAPI and FLA-PS (488) images. Additionally, these signals had to have a minimum overlap of 30%, a minimum area of 17 or 30 pixel (0.17–0.3 μm^2^) (DAPI signal and FLA-PS signal, respectively) and a minimum signal background ratio of 1 or 2.5 (DAPI and FLA-PS signals, respectively) (Bennke *et al.* 2016).

FISH was carried out with slight alterations of the protocol by Manz *et al.* (1992). The hybridisation buffer contained 900 mM NaCl, 20 mM Tris-HCl (pH 7.5), 0.02% sodium dodecyl sulphate, 10% dextran sulphate (wt/vol) and 1% (wt/vol) blocking reagent (Boehringer; Mannheim, Germany) with a formamide concentration optimised for individual probes (Supplementary Table S1). All hybridisations were carried out at 46 °C in a humidity chamber for 3 h, with a subsequent wash in a buffer containing 14–900 mM NaCl (dependent on formamide concentration in the hybridisation buffer), 20 mM Tris/HCl (pH 8), 5 mM EDTA (pH 8) and 0.01% sodium dodecyl sulphate at 48 °C. For super-resolution structured illumination microscopy (SR-SIM), the cells were initially scraped from the filter using a sterile scalpel and heat fixed to coverslips at 46 °C. After heat fixation, FISH was carried out as described above.

### Super-resolution structured illumination microscopy

Substrate incubation samples were visualised on a Zeiss ELYRA PS.1 (Carl Zeiss) using 561, 488 and 405 nm lasers and BP 573-613, BP 502-538 and BP 420-480+LP 750 optical filters. *Z*-stack images were taken with a Plan-Apochromat 63 × /1.4 Oil objective and processed with the software ZEN2011 (Carl Zeiss). SR-SIM images are taken by exciting the sample using non-uniform wide-field illumination. The laser light passes through an optical grating, generating a striped-shaped sinusoidal interference pattern. This pattern then combines with the sample information originating from structures below the diffraction limit to generate moire fringes. The image is detected by a CCD camera and contains high spatial frequency sample information shifted to a lower spatial frequency band that is transmitted through the objective. Mathematical reconstructions from raw image slices then allow for a reconstruction of a high-resolution image with doubled resolution in the *xy* plane (Schermelleh *et al.*, 2010). Intensity line profiles of individual cells were carried out using the ZEN black software (Carl Zeiss).

### Medium preparation

HaHa medium was prepared as described in detail by Hahnke and Harder (2013) and Hahnke *et al.* (2015). The basic HaHa medium was supplemented with 0.2 μm sterile filtered carbon sources (glucose, cellobiose, yeast extract, peptone, casamino acids (Hahnke and Harder, 2013), laminarin (Sigma-Aldrich) and FLA-laminarin) to make different carbon source media (Supplementary Table S2).

### Pure culture FLA-PS incubations

FLA-PS incubations were performed using a pure culture of *Gramella forsetii* strain KT0803 (DSM 17595), a marine member of *Bacteroidetes*. All growth experiments were carried out in biological duplicates. For all incubations and sampling time points (see below), cell growth and FLA-substrate uptake was analysed by fixing 1 ml of culture using 2% sterile filtered formaldehyde for 1 h at RT. Subsequently, the sample was filtered through a 25 mm polycarbonate filter (0.2 μm pore size), applying a gentle vacuum of <200 mbar, and the cells were visualised using microscopy (see ‘Substrate staining, FISH and epifluorescence microscopy’ section above). Cell fluorescence due to FLA-substrate uptake was quantified using an Accuri C6 flow cytometer (BD Accuri Cytometers, Ann Arbor, MI, USA).

*G*. *forsetii* was grown in HaHa high carbon medium (Supplementary Table S2) until it reached the stationary phase (48 h) and a cell count of 10^7^ cells ml^−1^. Subsequently, *G*. *forsetii* was inoculated (1:10) into HaHa minimal medium and grown for 48 h; this was repeated twice to starve the culture and mimic a minimal carbon environment. To track the uptake of FLA-laminarin by *G*. *forsetii*, in a low carbon environment, this starved culture was inoculated (1:10) into HaHa FLA-laminarin 35 μM medium and sampled every 3 h.

To allow for the upregulation of gene expression and production of enzymes capable of laminarin uptake (induction), the starved *G*. *forsetii* culture was inoculated into HaHa laminarin medium for 48 h (1 × 10^6^ cells ml^−1^). This was also carried out to test whether the uptake of FLA-laminarin increased after induction of the cells, mimicking an environment where laminarin is already available. Induced *G*. *forsetii* was inoculated (1:10) in both HaHa FLA-laminarin 35 μM medium and HaHa FLA-laminarin 3.5 μM medium. Substrate staining was tracked by sampling at 5, 20, 40, 60, 80 and 100 min.

To further analyse the FLA-substrate uptake by the cells and to determine whether the FLA tag can be excreted, after 100 min incubation in HaHa FLA-laminarin 35 μM medium, the substrate-stained cells were inoculated (1:10) in HaHa laminarin. The decrease in FLA signal was analysed by sampling at 20, 40, 60, 80, 100 min and 1 day after inoculation.

All growth experiments without FLA-laminarin were sampled regularly to check for autofluorescence or other sources of fluorescence that could be mistaken for substrate signals. Additionally, to ensure that there was no unspecific binding of FLA-laminarin to cells, cells grown in HaHa high carbon medium and HaHa laminarin medium were fixed using 2% sterile filtered formaldehyde for 1 h at RT and subsequently incubated with 35 μM FLA-laminarin for 4 h. The cells were then filtered through a 25 mm (0.2 μm pore size) polycarbonate filter, applying a gentle vacuum of <200 mbar and visualised using microscopy. There was no unspecific binding of substrate to fixed cells.

### Flow cytometry and fluorescence quantification

Cell fluorescence due to FLA-substrate uptake was quantified in all *G*. *forsetii* growth cultures using an Accuri C6 flow cytometer (BD Accuri Cytometers). Initially, the cells were fixed in 37% sterile filtered formaldehyde (final concentration 2%) for 1 h at RT. The 8- and 6-peak validation bead suspensions (Spherotech, Lake Forest, IL, USA) were used as internal references. The cells were analysed under laser excitation at 488 nm from a blue-green diode laser and the green fluorescence was collected in the FL1 channel (530±30 nm). An electronic threshold of 10 000 FSC-H was set to reduce background noise. All samples were analysed at the same flow rate (slow) and a total of 20 000 events per sample were acquired. Bacteria were detected from the signature plot of SSC-H vs green fluorescence (FL1-H). The FCM output was analysed using the BD Accuri software. Cells were assumed to give a positive signal if their associated mean fluorescence intensity was greater than the FL1-H of a culture not incubated in FLA-laminarin. As *G*. *forsetii* can form aggregates over time, a subset of data was defined using gates that represented single cells. For these gates, comparative fluorescence intensity was carried out by comparison of the mean fluorescence intensity to that of non-FLA-laminarin-stained cells.

## Results and discussion

**Figure 1:**
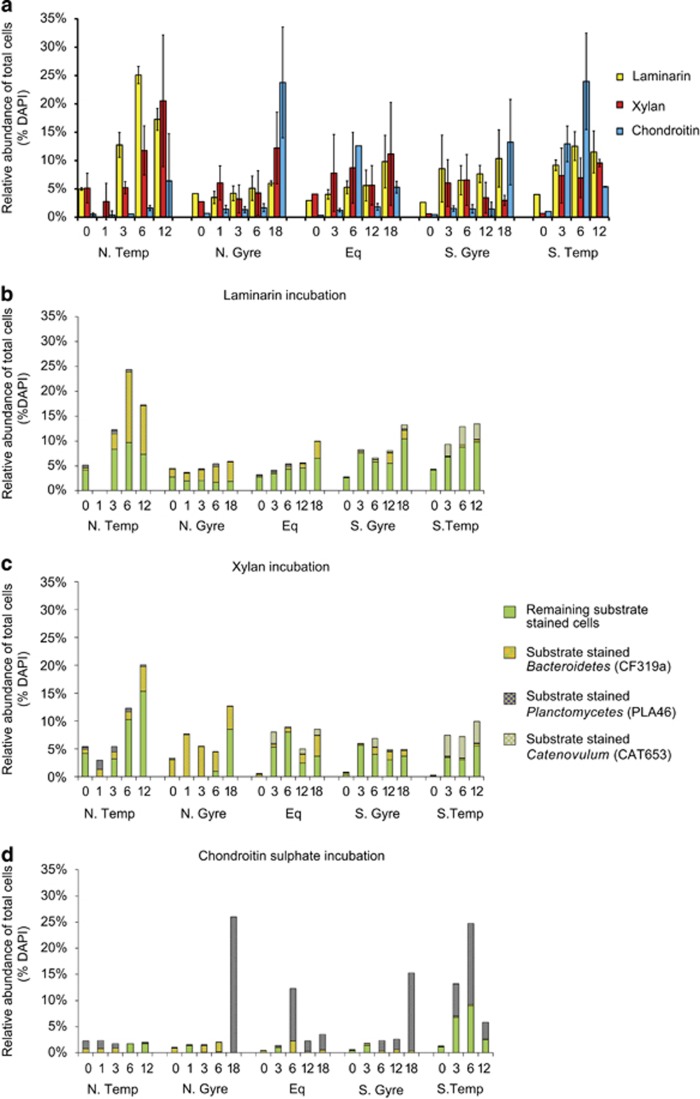
Relative abundance of substrate-stained cells and FISH identification of substrate-stained cells in incubations of seawater from five stations of different provinces of the Atlantic Ocean (see Supplementary Figure S1 for station locations). (**a**) relative abundance of cells stained by laminarin (yellow), xylan (red) and chondroitin sulphate (blue). Incubation time is indicated by ‘*T*’ (0–18 days). *T*0 refers to samples taken approximately 30 min after addition of the FLA-PS. (**b**) Relative abundance of laminarin-stained cells and fraction stained by FISH probes for *Bacteroidetes* (CF319a), *Planctomycetes* (PLA46) and *Catenovulum* (CAT653). (**c**) Relative abundance of xylan-stained cells and fraction stained by FISH probes for *Bacteroidetes* (CF319a), *Planctomycetes* (PLA46) and *Catenovulum* (CAT653). (**d**) Relative abundance of chondroitin-stained cells and fraction stained by FISH probes for *Bacteroidetes* (CF319a), *Planctomycetes* (PLA46) and *Catenovulum* (CAT653).

**Figure 2:**
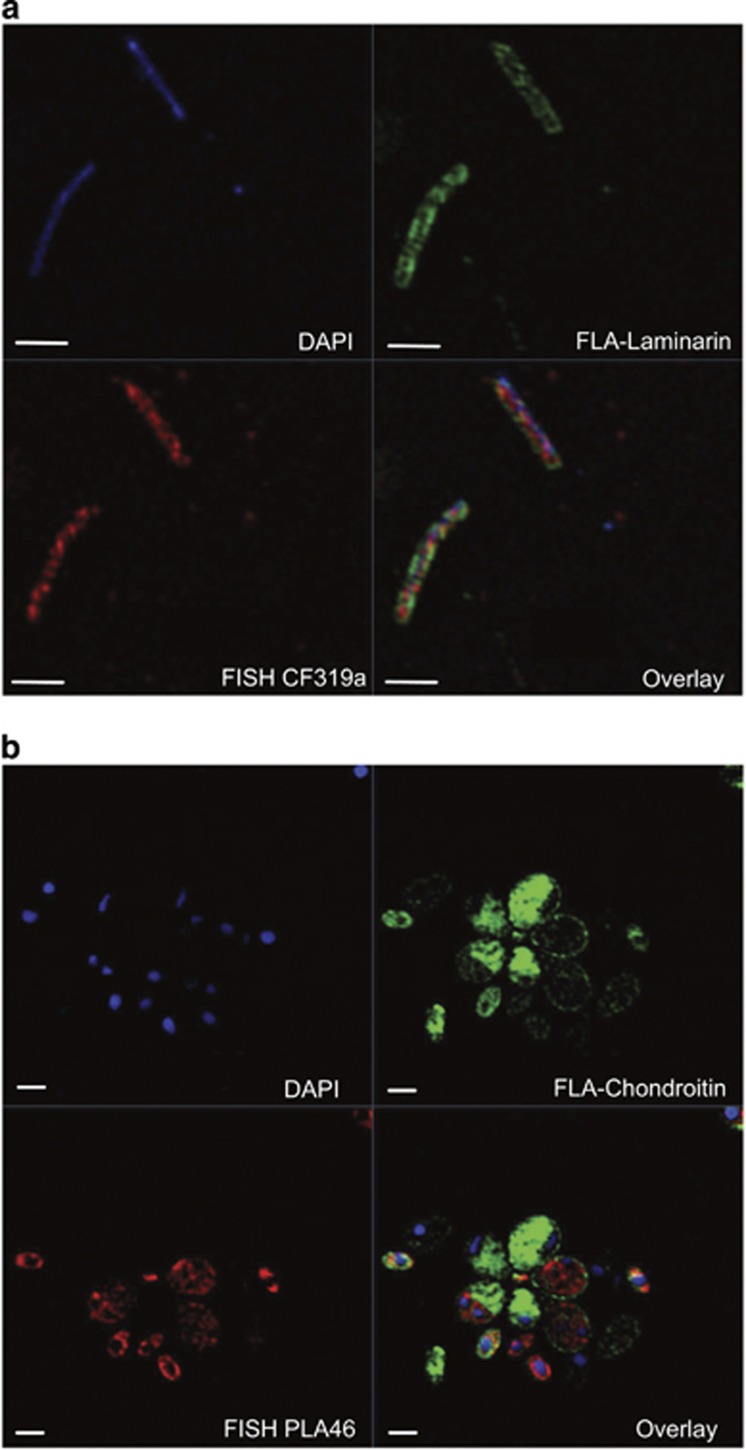
SR-SIM of cells stained by (**a**) DAPI (blue), FLA-laminarin (green) and *Bacteroidetes*-specific FISH probe (CF319a, red); (**b**) DAPI (blue), FLA-chondroitin sulphate (green) and *Planctomycetes*-specific FISH probe (PLA46, red). Scale bar=1 μm.

We incubated sea water from five distinct oceanic provinces with three FLA-PS and observed by epifluorescence microscopy that a considerable fraction of the bacterial community—up to 26% of total cells (Figure 1a)—bound detectable amounts of substrate (Figure 2,Supplementary Figure S2). Fluorescent staining was seen in all incubations but varied considerably with substrate and station (Figure 1a). The highest overall abundance and most rapid staining of cells was usually seen in the laminarin incubations. For example, in the Northern Temperate province 5% of cells show staining after just 30 min (*T*0) and the abundance increased to 25% after 6 days. Xylan staining of cells was also seen quickly (5% at *T*0) in the Northern Temperate, Northern Gyre and Equatorial stations but not in the Southern Gyre or Southern Temperate stations. The highest abundance of xylan-stained cells was in the Northern Temperate station after 12 days (20%). In the chondroitin sulphate incubations, the increase of substrate-stained cells occurred more slowly. High abundances of 22±3% were nonetheless observed at later time points (*T*18) in the gyre stations. At the Southern Temperate station, a high percentage of cells were stained already at *T*6. Many different cell morphologies were observed among substrate-stained cells—coccoid, rod shaped and ovoid; the rate of staining of the different cell types varied (Figure 2,Supplementary Figure S2). A diverse range of bacteria thus were binding FLA-PS.

Examination of individual cells using SR-SIM, which enables visualisation of prokaryotic cell compartments, showed that the initial association of cells with substrates occurred in the cell periphery within 30 min (Supplementary Figure S4a). Such rapid direct staining was surprising, as polysaccharide additions were moderate (ca. 21 μM C) and the average number of fluorophores per molecule of polysaccharide (the labelling density) was low (between 0.5 and 1.3 per polysaccharide molecule; Arnosti (2003). Currently, it is not possible to measure the absolute number of fluorophores taken up over time by individual cells in an environmental sample. However, a comparison of the FLA-PS signal and the FISH signal resulting from a 4 × labelled rRNA targets oligonucleotide of the same cell (Figure 2) suggests that thousands of FLA-PS molecules have been bound by individual cells.

Rapid staining was also observed in pure cultures of *G*. *forsetii* incubated with FLA-laminarin (Supplementary Figure S3 and Supplementary Table S3). When *G*. *forsetii* cells grown on a minimal carbon medium with no polysaccharide were inoculated into a medium containing FLA-laminarin (35 μM monomer l^−1^), it took up to 12 h for substrate-specific staining to be observed. However, when *G*. *forsetii* was grown on a laminarin medium and subsequently inoculated into FLA-laminarin (3.5 μM monomer l ^−1^) containing media, staining could be seen within minutes (Supplementary Figure S3 and Supplementary Table S3). This result not only shows the high affinity of induced *G*. *forsetii* towards laminarin but also demonstrates that a fraction of the environmental bacteria were likely induced or specialised for the immediate uptake of polysaccharides, as seen by staining at *T*0 (Figure 1a and Supplementary Figure S4).

Live *G*. *forsetii* lost much of the substrate signal from FLA-laminarin within 24 h (Supplementary Table S3). Specifically, when *G*. *forsetii* was transferred from HaHa FLA-laminarin medium into HaHa laminarin medium, cells continued growing and simultaneously lost the FLA-laminarin signal over time. The slow removal of FLA from the cell indicates that the substrate was not unspecifically bound to the cell surface but instead taken up into the cell. Moreover, the loss of signal in *G*. *forsetii* over time cannot be solely related to dilution through cell division, as the signal decreased more rapidly than average doubling times of *G*. *forsetii*.

In environmental samples, the overall abundance of substrate-stained cells increased with time in all incubations (*R*^2^=0.0823, *P*-value=0.0153) (Figure 1a), despite the fact that (with a single exception) the absolute cellular abundances within the incubations did not increase significantly (Supplementary Figure S5). Although the abundance of stained cells increased, this relationship varied by station and substrate. At the Equatorial station (xylan and chondroitin sulphate incubations) and the Southern Gyre station (laminarin and xylan incubations), moreover, there was a decrease in the abundance of substrate-stained cells between day 3 and day 6. No substrate staining was detected in heat-killed or formaldehyde-fixed cell controls, indicating that staining was due to biological activity (Supplementary Figure S6).

The ability to use polysaccharides has been confirmed for many marine bacterial phyla, including *Bacteroidetes*, *Planctomycetes*, *Verrucomicrobia* and *Proteobacteria* (Martinez-Garcia *et al.*, 2012; Teeling *et al.*, 2012; Kabisch *et al.*, 2014; Lage and Bondoso, 2014; Wietz *et al.*, 2015). Based on the literature (Schattenhoffer *et al.*, 2009; Teeling *et al.*, 2012; Lage and Bondoso, 2014; Wietz *et al.*, 2015) and cell morphologies, a selection of group-specific FISH probes (Supplementary Table S1) was used to identify and enumerate specific bacterial group abundances at each time point and station. The combination of FISH with substrate staining allowed for the identification of organisms directly taking up a specific substrate (Figures 2a and b). Using this probe set (CF319a, PLA46 and CAT653 targeting the *Bacteroidetes*, *Planctomycetes* and *Catenovulum*, respectively), an average of 48%±49% (median of 55%) of the substrate-stained cells could be identified (Figures 1b–d). Future analysis using 16S rRNA sequencing is currently being pursued to supplement this probe set in future and further increase the fraction of identified cells.

The FISH counts of the substrate incubations of the Atlantic Ocean showed an increase in abundance of the selected clades. For example, FISH counts of the laminarin incubation at the Northern Temperate station showed a nearly fivefold increase in *Bacteroidetes* abundance over 6 days (from 7.2 × 10^4^ to 3.3 × 10^5^ cells ml^−1^ at day 6), with 80% of these cells showing substrate-specific staining (Figure 1b and Supplementary Figure S9). In the other regions, *Bacteroidetes* did not increase as strongly in abundance, possibly due to lower initial cell numbers, but the percentage of substrate stained *Bacteroidetes* increased from 5% to 72±28% in 12–18 days.

*Planctomycetes* cellular abundances likewise increased, particularly in the chondroitin sulphate incubation of the Southern Temperate station, from 2.3 × 10^3^ to 2.5 × 10^5^ cell ml^−1^ over 6 days; 84% of these cells showed substrate staining (Figure 1d and Supplementary Figure S9). The substrate-stained cells in the chondroitin sulphate incubations of other regions exhibited distinctive *Planctomycetes-*like cell morphologies but were only partially identified as *Planctomycetes* with FISH probe PLA46, perhaps due to difficulty of permeabilising *Planctomycetes* cells (Pizzetti *et al.*, 2011). In addition to the *Bacteroidetes* and *Planctomycetes*, the gammaproteobacterial genus *Catenovulum* also showed substrate-specific staining. *Catenovulum* increased in abundance in the laminarin and xylan incubations at the Equatorial, Southern Gyre and Southern Temperate stations and constituted 1–4% of the total stained cells.

**Figure 3:**
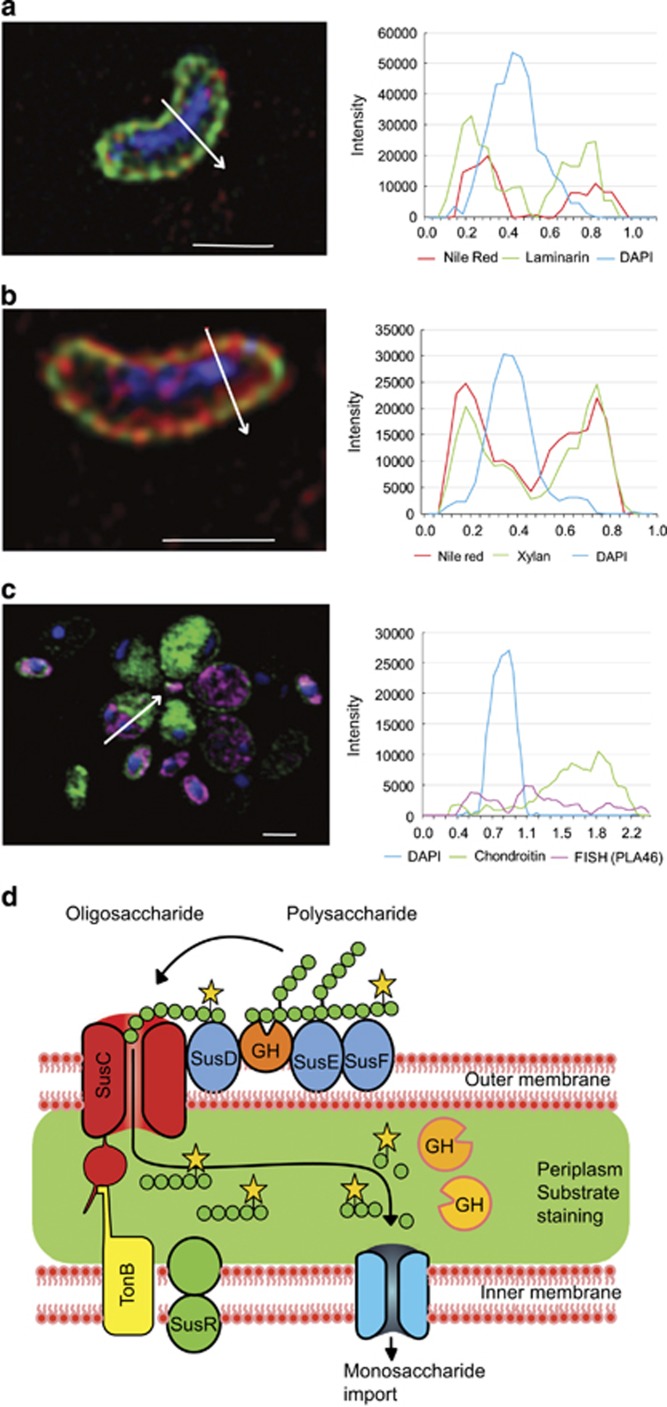
SR-SIM images showing Sus-like uptake of FLA-PS and fluorescence intensity line profiles localising substrate-specific staining after 6 days of incubation. (**a** and **b**) Cells stained with FLA-PS (green), nile red (red; membrane) and DAPI (blue; DNA). White arrows indicate sections along which the fluorescence intensity line profiles were recorded. Scale bars=1 μm. Corresponding profiles indicating co-localisation of substrate and membrane are shown on the right. (**a**) *Bacteroidetes* cell from the Northern Temperate station stained with FLA-laminarin. (**b**) *Bacteroidetes* cell from the Southern Temperate station stained with FLA-xylan. (**c**) *Planctomycetes* cells from the Southern Temperate station stained with FLA-chondroitin. Cells were identified using the FISH probe PLA46 (magenta), which labels the riboplasm; the substrate staining is in the paryphoplasm. (**d**) Conceptual model of sus-like bacterial uptake of FLA-PS into the periplasm via TonB-dependent outer membrane transporters, causing halo-like staining in periplasm (green). The large oligosaccharides are further hydrolysed within the periplasm to monosaccharides, disaccharides or trisaccharides, which are subsequently transported into the cytoplasm. Modified after Koropatkin *et al.* (2012). ‘GH’ represents glycoside hydrolases.

Substrate-stained cells predominantly exhibited a halo-like staining that was restricted to the cell periphery (Figures 3a and b,Supplementary Figure S8). Fluorescence intensity line profiles of individual cells in combination with nile red membrane counterstaining showed a co-localisation of the substrate signal with the cell membranes (Figures 3a and b). However, in cells <0.5 μm in width, for example, some *Bacteroidetes*, the halo-like staining could not, due to limits of optical resolution, definitively be shown to be within the periplasmic space between the membranes. In contrast, for the larger cells of *Planctomycetes* uptake of FLA-chondroitin sulphate across the outer membrane could be seen (Figure 3c and Supplementary Figure S7). SR-SIM revealed that FLA-chondroitin sulphate had been transported into the paryphoplasm but not into the riboplasm (Figure 3c and Supplementary Figure S7). Similar to the periplasm of Gram-negative bacteria, the paryphoplasm is a cell compartment located between an inner and an outer membrane that does not contain ribosomes (Fuerst and Sagulenko, 2011).

Genomic analyses of marine strains from *Bacteroidetes* and *Planctomycetes* have shown that they have the potential to express carbohydrate transporters for the uptake of large oligosaccharides through the outer membrane (Fuerst and Sagulenko, 2011; Teeling *et al.*, 2012; Paparoditis *et al.*, 2014). Intestinal *Bacteroidetes* in particular are known for sus-like polysaccharide utilisation loci (Koropatkin *et al.*, 2012). When expressed, the proteins encoded in these loci are principally located in the outer membrane and the periplasm. They sequentially bind and hydrolyse polysaccharides, transporting large oligosaccharides into the periplasm, where further degradation occurs in a protected space (Figure 3d; Koropatkin *et al.*, 2012). Our observations of the staining patterns of marine *Bacteroidetes* cells are consistent with this mode of substrate processing. Previous research on *G*. *forsetii* has shown that, in laminarin-amended cultures, the expression of proteins required for its binding, transport and utilisation of laminarin is induced (Kabisch *et al.*, 2014). Here we show that induced *G*. *forsetii* cells are stained with FLA-laminarin in minutes (Supplementary Figure S3), whereas cells that were not induced required hours before staining can be detected. Up to 5% of open ocean bacteria are readily stained with FLA-laminarin, showing detectable staining after just 30 min (*T*0), which indicates that for these bacteria no induction is required: they are primed for rapid uptake of laminarin into the periplasm. (Supplementary Figure S4a).

Based on our microscopic examination of *Bacteroidetes* from the surface ocean, data from pure cultures of *G*. *forsetii* and genomic information (Koropatkin *et al.*, 2012; Teeling *et al.*, 2012; Kabisch *et al.*, 2014), we hypothesise that the substrate uptake we observed is homologous to the sus-like mechanism of gut *Bacteroidetes*. This mechanism has recently been referred to as ‘selfish’ by Cuskin *et al.* (2015) owing to the fact that, after uptake of large oligosaccharides, further degradation occurs in the protected periplasmic space. Selfish substrate uptake confers a distinct ecological benefit by minimising formation of monosaccharides, disaccharides and trisaccharides in the external environment and avoiding diffusive loss of enzymes (Figure 3d; Cuskin *et al.*, 2015). The uptake of chondroitin sulphate by *Planctomycetes* likely occurs via an analogous but unknown mechanism.

As demonstrated by the substantial fraction of the natural microbial community that was stained by just three distinct substrates (Figure 1a), this substrate utilisation strategy is important not only in the anaerobic and organic-carbon rich environment of the human gut (Cuskin *et al.*, 2015) but also in the oxic, dilute and comparatively organic-carbon-poor surface waters of the Atlantic Ocean. Although these experiments were carried out in bottle incubations that may well have led to changes in the microbial community from its initial composition, the organisms growing later in the incubations must ultimately have originated from the seawater sample we collected. The activity seen later in the incubations thus may not reflect the exact dynamics that occur in the ocean, but nonetheless highlight the selfish uptake capabilities that these microorganisms possess. Moreover, the observation that FLA-PS were bound by organisms in both the early (initial 30 min) and late (up to 18 days) phases of the incubations suggest that this strategy of substrate acquisition competes well with alternative strategies of substrate utilisation in complex microbial communities. These observations imply that a re-evaluation of models of bacterial substrate utilisation in natural environments will be necessary. Current models typically encompass two classes of organisms, those that produce enzymes that release low-molecular-weight substrates to the environment and those that use the hydrolysis products but do not produce enzymes themselves (for example,Allison, 2005; Kaiser *et al.*, 2015; Traving *et al.*, 2015). These two-player models will need to be expanded to consider organisms that have evolved mechanisms to minimise substrate sharing.

## Conclusions

Measurements and models of the manner in which the most abundant products of photosynthesis, polysaccharides, are channelled into the microbial food chain need to account for the varying ecological strategies of heterotrophic marine bacteria. Most field measurements of enzyme activities rely on substrate proxies containing monomers (Zaccone *et al.*, 2012; Kellogg and Deming, 2014); carbohydrate uptake measurements in ocean waters likewise are made most frequently with monosaccharides (Rich *et al.*, 1996). These measurements do not account for bacteria that quickly capture and process HMW polysaccharides. The speed and extent of selfish substrate uptake by phylogenetically distinct bacteria at five widely spaced stations in the Atlantic Ocean demonstrate that this is an important mechanism of carbon utilisation that previously has been overlooked. Future models as well as measurements will need to account for this mode of substrate acquisition as part of microbially driven carbon cycling in the ocean.

The FLA-PS staining method, in combination with FISH, allows for direct identification of polysaccharide-degrading bacteria in environmental samples. Based on this new method, future studies can specifically measure the types and quantities of phytoplankton-produced polysaccharides that are processed by this mechanism, as well as explore other locations and conditions under which selfish substrate utilisation may predominate.
