# Supplementary material for: An alternative polysaccharide uptake mechanism of marine bacteria
Source: ISME J. 2017 Mar 21;11(7):1640–50. doi: 10.1038/ismej.2017.26 (PMC5520146; doi:10.1038/ismej.2017.26)
Supplement: Supplementary Table S1 [file ismej201726x1.doc]

Table S1 | FISH probes used in this study

| Probe Name | Sequence 5' - 3' | FA% | Reference |
| --- | --- | --- | --- |
| CF319a  PLA46  CAT653 | TGGTCCGTGTCTCAGTAC  GACTTGCATGCCTAATCC  CCCCCTCTCCCTTACTCT | 35  30  25 | Manz *et al.*(1992)  Neef et al. (1998)  This study |

**FA corresponds to the formamide concentration using in the hybridisation buffer.*
