# Supplementary material for: An alternative polysaccharide uptake mechanism of marine bacteria
Source: ISME J. 2017 Mar 21;11(7):1640–50. doi: 10.1038/ismej.2017.26 (PMC5520146; doi:10.1038/ismej.2017.26)
Supplement: Supplementary Table S2 [file ismej201726x2.doc]

Table S2. Carbon source and concentration added to individual media.

| Medium | Substrate | Stock concentration | Volume added [ml] to 1L |
| --- | --- | --- | --- |
| HaHa high carbon | Glucose, cellobiose, yeast extract, peptone and casamino acids | 100 g l^-1^ | 2 ml |
| Ha Ha minimal | Glucose, cellobiose, yeast extract, peptone and casamino acids | 1 g l^-1^ | 1.2 ml |
| HaHa Laminarin | Yeast extract and laminarin | 1 g l^-1^ and 50g l^-1^ | 1.2 ml and 1 ml |
| HaHa FLA-Laminarin 3.5µM | FLA-Laminarin | 16.6 mM monomer l^-1^ | 0.021 ml |
| HaHa FLA-Laminarin 35µM | FLA-Laminarin | 16.6 mM monomer l^-1^ | 0.210 ml |
