# Supplementary material for: An alternative polysaccharide uptake mechanism of marine bacteria
Source: ISME J. 2017 Mar 21;11(7):1640–50. doi: 10.1038/ismej.2017.26 (PMC5520146; doi:10.1038/ismej.2017.26)
Supplement: Supplementary Table S3 [file ismej201726x3.doc]

Table S3 | Flow cytometry results showing signal intensities (FL1 533/30.H) from cells incubated with varying FLA-laminarin concentrations over time (5 - 100 min). Background shows the FL1 signal intensity of *G. forsetii* without the addition of FLA-laminarin. The substrate loss signal indicates the reduction of the FL1 signal after 1 day after inoculation (1:10) into HaHa laminarin medium.

| Substrate Concentration (µM) | Incubation time (min) | | | |  | Substrate Loss | |
| --- | --- | --- | --- | --- | --- | --- | --- |
|  | 5 | 20 | 40 | 60 | 100 | 1260 | |
| 35 | 2 373 | 2 194 | 2 107 | 2 061 | 1 919 | 501 |  |
| 15 | 1 670 | 1 462 | 1 420 | 1 363 | 1 325 |  |  |
| 5 | 1 178 | 1 005 | 942 | 894 | 819 |  |  |
| 3.5 | 1 108 | 916 | 876 | 814 | 759 |  |  |
| 1 | 803 | 579 | 525 | 473 | 426 |  |  |
| 0.5 | 371 | 254 | 241 | 232 | 219 |  |  |
| 0.05 | 291 | 218 | 211 | 207 | 203 |  |  |
| 0  (Background) | 179 |  |  |  |  |  |  |
