# Supplementary material for: An alternative polysaccharide uptake mechanism of marine bacteria
Source: ISME J. 2017 Mar 21;11(7):1640–50. doi: 10.1038/ismej.2017.26 (PMC5520146; doi:10.1038/ismej.2017.26)
Supplement: Supplementary Figure Legends [file ismej201726x4.pdf]

Supplementary Figure Legends

Supplementary Figure S1. Sampling sites in the Atlantic Ocean. (a) Sites shown by white dots; background colours indicate the average chlorophyll *a* concentration (mg m^{-3}) during the sampling time period (map obtained from MODIS, Ocean Biology Processing Group (2014). (b) Latitude and longitude of each sampling site.

Supplementary Figure S2. Epifluorescence microscopy images showing the morphological variability of substrate stained cells. Images were taken from the laminarin incubation of the Northern Temperate station after 3 days (above) and the chondroitin sulphate incubation of the Southern Temperate station after 6 days (below). Cells were stained by DAPI to visualise the DNA (left, blue) and show FLA-laminarin or FLA-chondroitin sulphate specific staining (middle, green). Scale bar = 5 µm.

Supplementary Figure S3. Detection of FLA-laminarin uptake by *G. forsetii* over time using flow cytometry. (a) Untreated control culture of *G. forsetii* showing background FL1-H signal (green gating). (b) *G. forsetii* incubated with 35 µM FLA-laminarin showing high FL1-H signal (green gating) after just 5 min. (c) *G. forsetii* incubation with 1 µM FLA laminarin showing low FLA signal after 5 min. (d and e) Substrate uptake by *G. forsetii* over time. SR-SIM images showing FLA-laminarin uptake by *G. forsetii* after 5 min and 100 min. Scale bar = 5 µm

Supplementary Figure S4. Change in substrate staining of cells over time. (a-d) Two colour (blue = DAPI, green = FLA-laminarin) super-resolution structured illumination microscopy images of cells from the Northern Temperate station incubated with fluorescently labelled laminarin. Time series incubation after (a) 30 min, (b) 3 days, (c) 6 days, and (d) 12 days. Intensity of staining increased over time. Scale bar = 1 µm.

Supplementary Figure S5. Change in absolute cellular abundance (cell ml $^{-1}$) during each substrate incubation (laminarin, xylan, chondroitin) and unamended treatment control over time in the Northern Temperate, Northern Gyre, Equatorial, Southern Gyre, and Southern Temperate station. The error bars indicate the total range of triplicate incubations.

Supplementary Figure S6. Panels (a) – (h) show epifluorescence microscopy images of DAPI stained cells excited by 365 nm with a constant exposure time of 30 ms (left side), and corresponding image of the same field of view excited by 488 nm at constant exposure of 300 ms (right side). Cells excited by 488 nm show in panel (b) positive FLA-laminarin signal in the Northern Temperate Station at T6, in panels (d) and (f) treatment control of the Northern Temperate and Southern Gyre station at T6. White scale bar = 10µm. Image (g) shows DAPI stained *G. forsetii* cell after fixation in 2% formaldehyde (FA) for 2 h and subsequent addition to FLA-laminarin medium for 2 h. Panel (h) is the corresponding image excited by 488 nm showing no unspecific substrate staining of cells. Red scale bar = 2 µm..

Supplementary Figure S7. SR-SIM Z-stack of cells incubated with chondroitin sulphate. Images (a-p) show horizontal slices of the cells at 0.2µm intervals. Chondroitin (green) is in the paryphoplasm; FISH signal (red, Pla46) is in the riboplasm, and DNA is stained by DAPI (blue). Scale bar = 1µm.

Supplementary Figure S8. Sus-like uptake of FLA-PS by *Bacteroidetes*. SR-SIM images of halo-like substrate staining (FLA-laminarin; green) in the Northern Temperate station after 6 days. Cell was counter stained using nile red (red) and DAPI (blue) to visualise the membranes and DNA, respectively. Scale bar = 0.5 µm.

Supplementary Figure S9. Absolute abundance of *Bacteroidetes, Planctomycetes, and Catenovulum* during substrate incubations and treatment control (a) laminarin, (b) xylan, (c) chondroitin sulphate, (d) treatment control) of the Northern Temperate, Northern Gyre, Equatorial, Southern Gyre, and Southern Temperate stations. The absolute abundance was determined by using group specific FISH probes CF319a (orange), PLA46 (purple) and CAT653 (pink) for *Bacteroidetes, Planctomycetes, and Catenovulum* respectively. Also shown is the percentage of *Bacteroidetes* (orange with green strips), *Planctomycetes* (purple with green strips) and *Catenovulum* (pink with green strips) showing substrate stained.
