# Supplementary material for: An alternative polysaccharide uptake mechanism of marine bacteria
Source: ISME J. 2017 Mar 21;11(7):1640–50. doi: 10.1038/ismej.2017.26 (PMC5520146; doi:10.1038/ismej.2017.26)
Supplement: Supplementary Figure S1 [file ismej201726x5.pdf]

Reintjes et al. Supplementary Figure S1

a

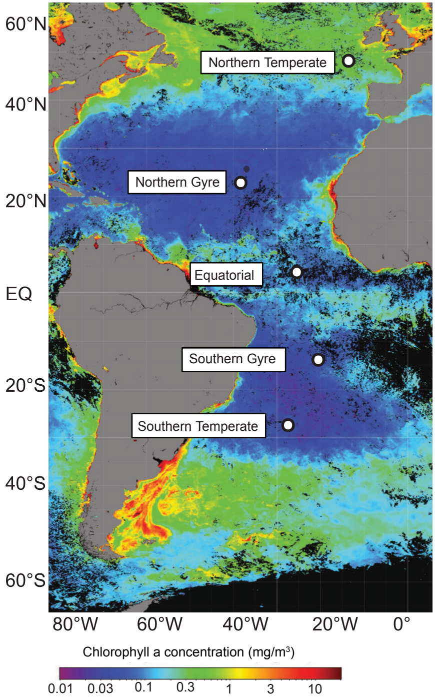

b

Site	Latitude	Longitude
Northern Temperate	47°52N	17°16W
Northern Gyre	22°04N	39°47W
Equatorial	2°55N	25°39W
Southern Gyre	11°37S	25°10W
Southern Temperate	26°57S	25°00W
