# Supplementary figures and images for: An alternative polysaccharide uptake mechanism of marine bacteria
Source: ISME J. 2017 Mar 21;11(7):1640–50. doi: 10.1038/ismej.2017.26 (PMC5520146; doi:10.1038/ismej.2017.26)

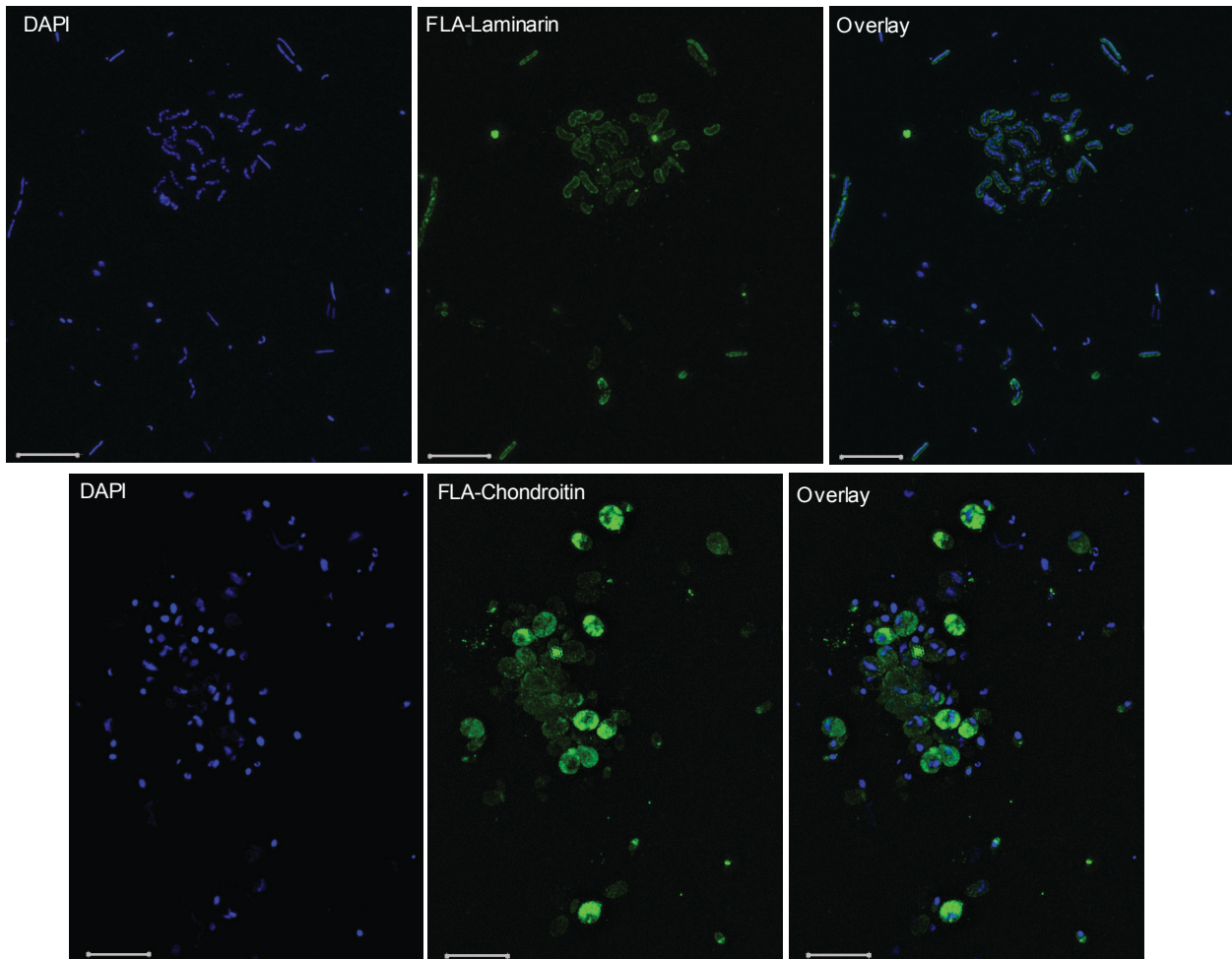

Supplement: Supplementary Figure S2 [file ismej201726x6.pdf]

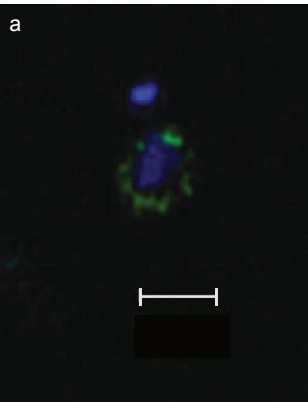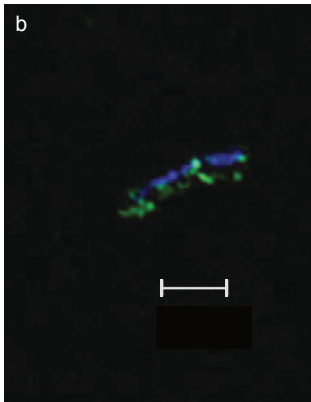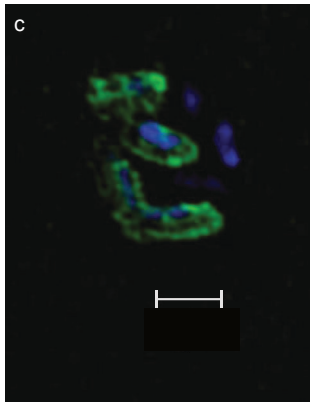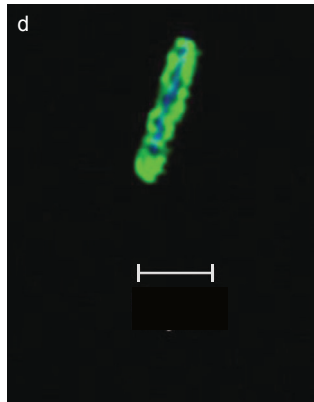

Supplement: Supplementary Figure S4 [file ismej201726x8.pdf]

Reintjes et al. Supplementary Figure S5

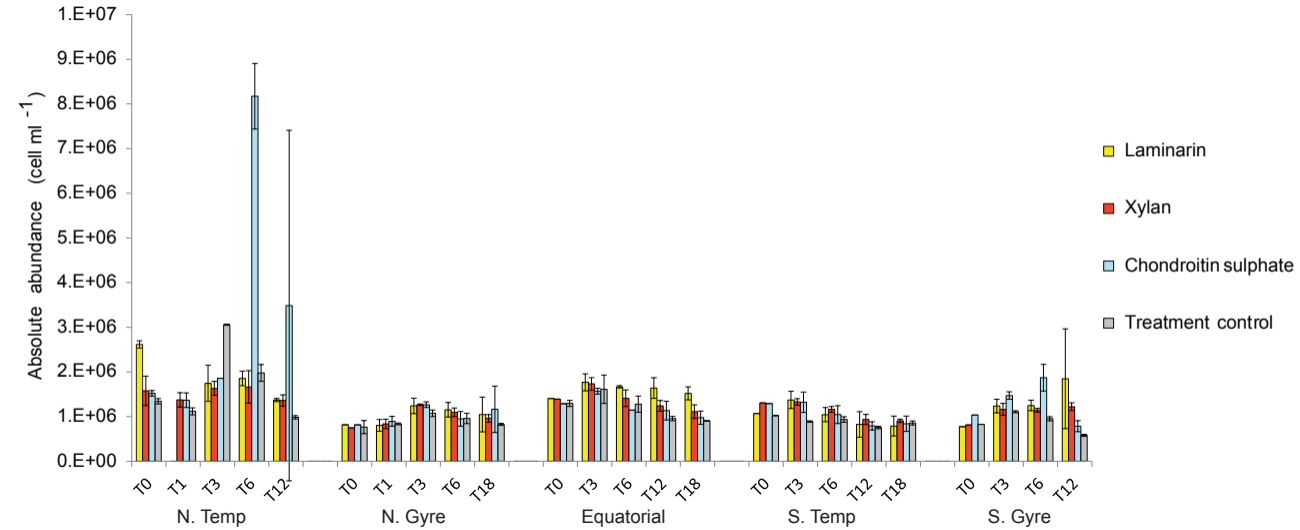

Supplement: Supplementary Figure S5 [file ismej201726x9.pdf]

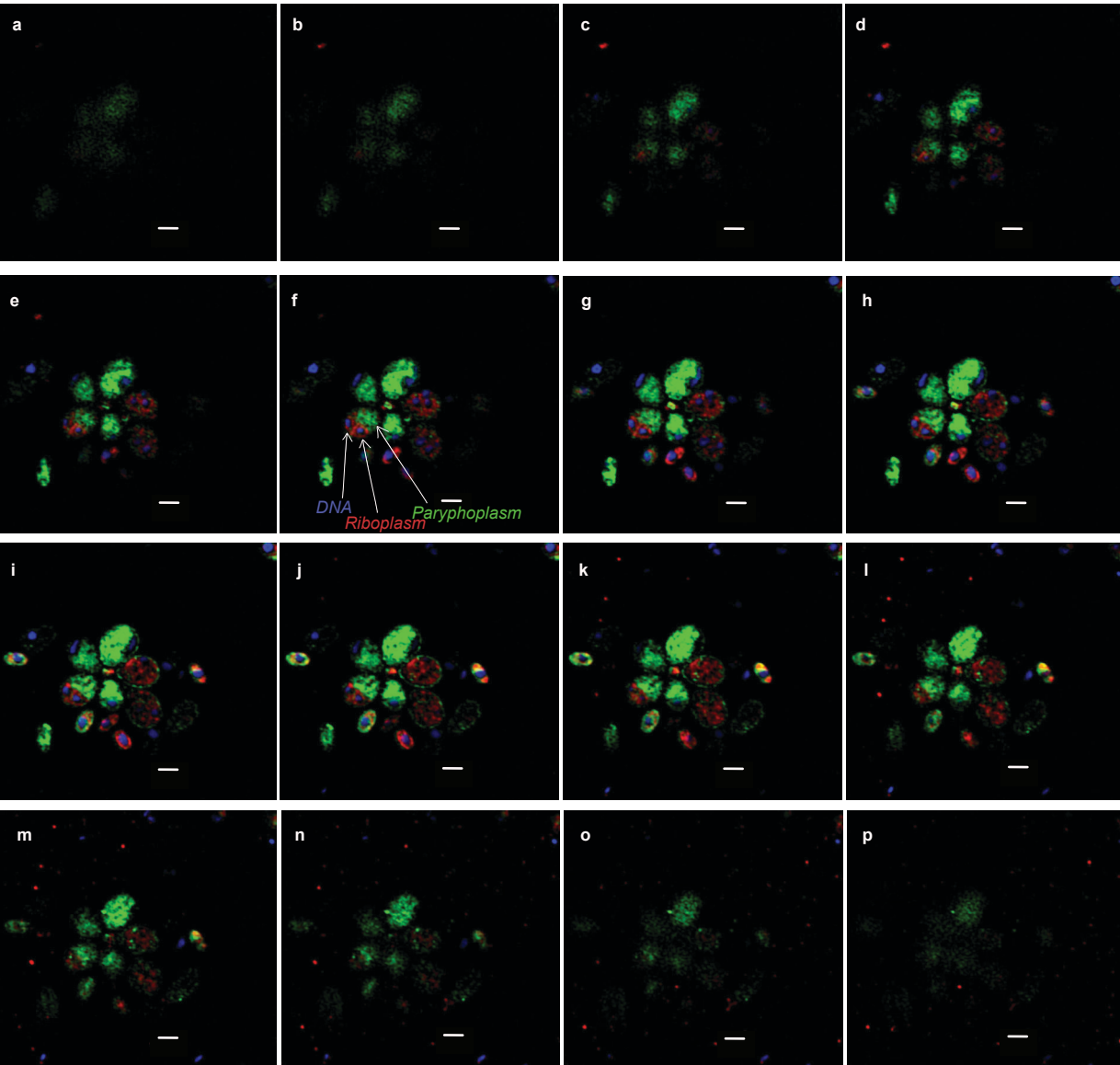

Supplement: Supplementary Figure S7 [file ismej201726x11.pdf]

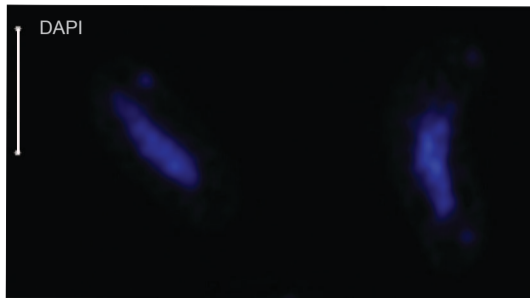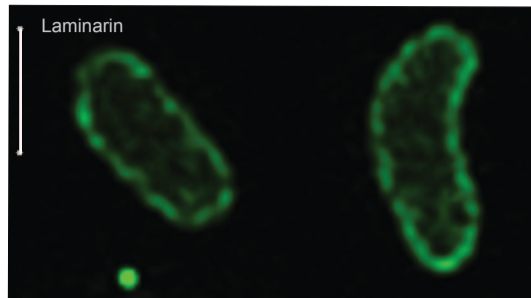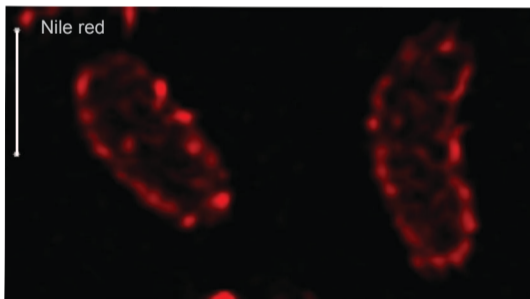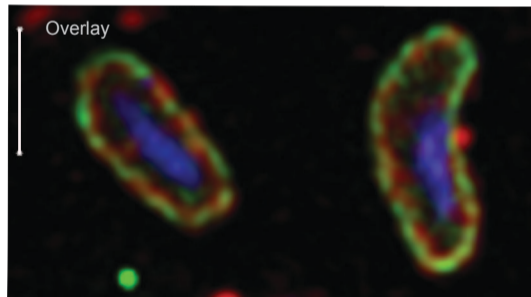

Supplement: Supplementary Figure S8 [file ismej201726x12.pdf]

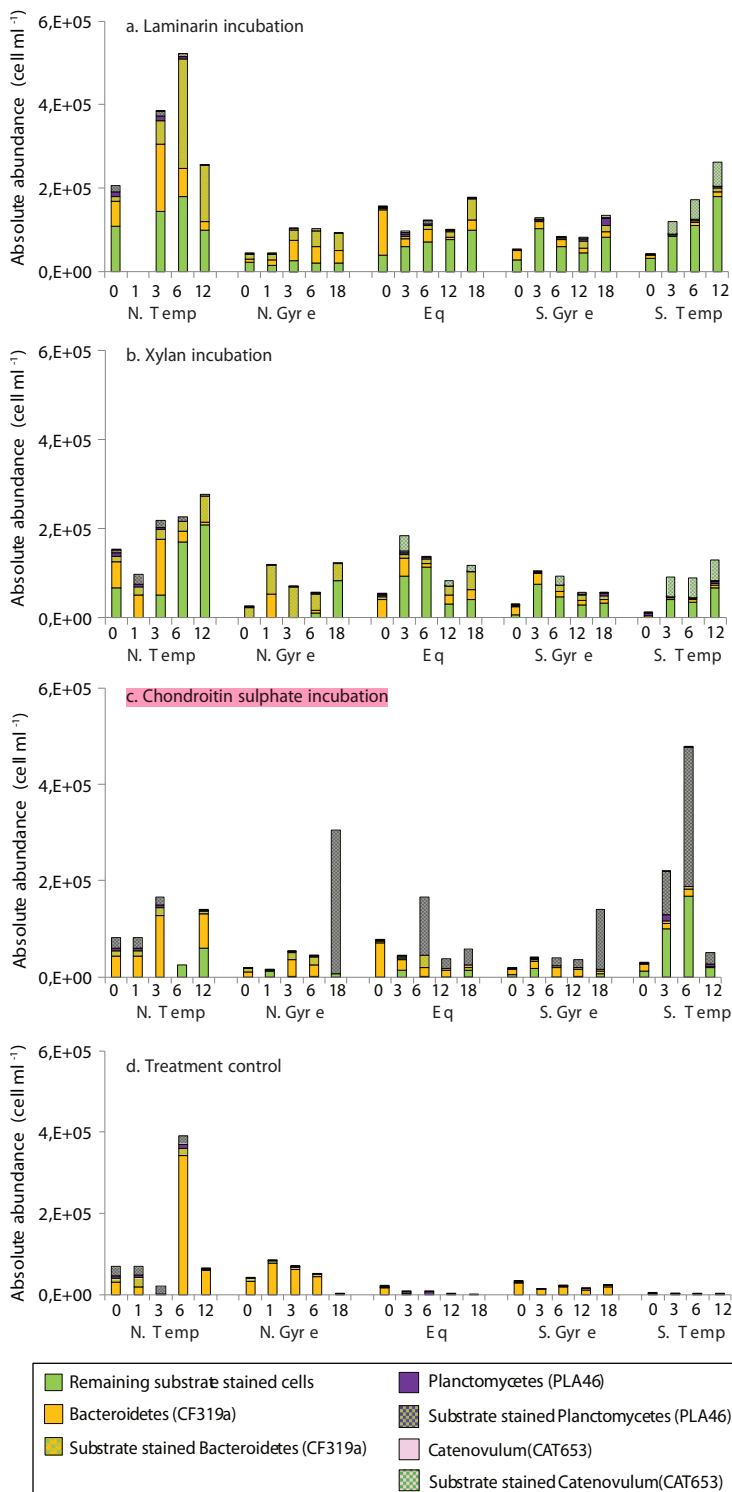

Supplement: Supplementary Figure S9 [file ismej201726x13.pdf]
