# Supplementary material for: An alternative polysaccharide uptake mechanism of marine bacteria
Source: ISME J. 2017 Mar 21;11(7):1640–50. doi: 10.1038/ismej.2017.26 (PMC5520146; doi:10.1038/ismej.2017.26)

Reintjes et al. Supplementary Figure S6

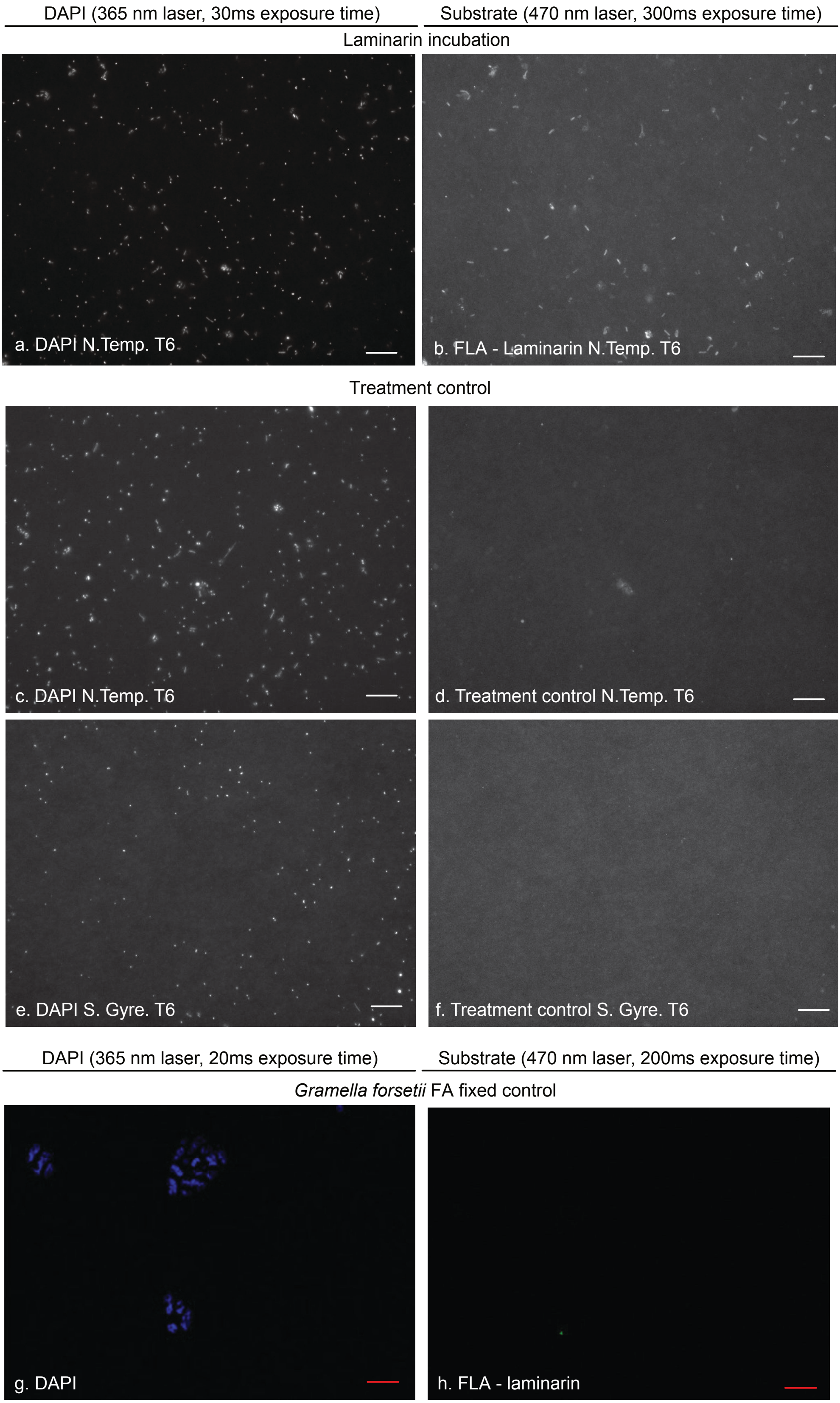

Supplement: Supplementary Figure S6 [file ismej201726x10.pdf]
